# Growth/Differentiation Factor-15 (GDF-15): From Biomarker to Novel Targetable Immune Checkpoint

**DOI:** 10.3389/fimmu.2020.00951

**Published:** 2020-05-19

**Authors:** Jörg Wischhusen, Ignacio Melero, Wolf Herman Fridman

**Affiliations:** ^1^Experimental Tumor Immunology, Department of Obstetrics and Gynecology, University of Würzburg Medical School, Würzburg, Germany; ^2^Program of Immunology and Immunotherapy, Center for Applied Medical Research (CIMA), Pamplona, Spain; ^3^Centro de Investigación Biomédica en Red Cáncer, CIBERONC, Madrid, Spain; ^4^Immunology and Immunotherapy Unit, Clínica Universidad de Navarra, Pamplona, Spain; ^5^INSERM, UMR_S 1138, Cordeliers Research Center, Université de Paris, Sorbonne Université Team Cancer, Immune Control and Escape, Paris, France

**Keywords:** growth/differentiation factor-15 (GDF-15), macrophage inhibitory cytokine-1 (MIC-1), cancer, pregnancy, autoimmunity, anorexia, immune exclusion, immunotherapy

## Abstract

Growth/differentiation factor-15 (GDF-15), also named macrophage inhibitory cytokine-1, is a divergent member of the transforming growth factor β superfamily. While physiological expression is barely detectable in most somatic tissues in humans, GDF-15 is abundant in placenta. Elsewhere, GDF-15 is often induced under stress conditions, seemingly to maintain cell and tissue homeostasis; however, a moderate increase in GDF-15 blood levels is observed with age. Highly elevated GDF-15 levels are mostly linked to pathological conditions including inflammation, myocardial ischemia, and notably cancer. GDF-15 has thus been widely explored as a biomarker for disease prognosis. Mechanistically, induction of anorexia via the brainstem-restricted GDF-15 receptor GFRAL (glial cell-derived neurotrophic factor [GDNF] family receptor α-like) is well-documented. GDF-15 and GFRAL have thus become attractive targets for metabolic intervention. Still, several GDF-15 mediated effects (including its physiological role in pregnancy) are difficult to explain via the described pathway. Hence, there is a clear need to better understand non-metabolic effects of GDF-15. With particular emphasis on its immunomodulatory potential this review discusses the roles of GDF-15 in pregnancy and in pathological conditions including myocardial infarction, autoimmune disease, and specifically cancer. Importantly, the strong predictive value of GDF-15 as biomarker may plausibly be linked to its immune-regulatory function. The described associations and mechanistic data support the hypothesis that GDF-15 acts as immune checkpoint and is thus an emerging target for cancer immunotherapy.

## Introduction

Growth/differentiation factor 15 (GDF-15) is also known as macrophage inhibitory cytokine (MIC)-1, non-steroidal anti-inflammatory drug-inducible gene (NAG)-1, placental transforming growth factor-beta (pTGFB), prostate-derived factor (PDF), and placental bone morphogenetic protein (PLAB). GDF-15 is a divergent member of the transforming growth factor-β (TGF-β) superfamily (1–3). It contains seven conserved cysteine residues forming a cysteine knot that is the defining hallmark of the TGF-β superfamily. Among all superfamily members, orthologous GDF-15 molecules show the lowest sequence conservation across species. While mature rat, mouse and human TGF-β1 and BMP-2 proteins are 99–100% sequence identical between species, homology is below 70% for GDF-15 (4). Moreover, promoter regions are entirely different between humans and mice (5).

Outside reproductive organs, GDF-15 shows low to absent constitutive expression; however, in many cell types it can be induced under stress conditions. The 308 amino acid chain comprises a signal peptide (29 amino acids), a pro-domain (167 amino acids) and the mature GDF-15 of 112 amino acids. While intracellular proteolytic processing is possible, GDF-15 is mostly secreted as pro-protein, and the pro-domain remains attached to the extracellular matrix (ECM). Thus, latent stromal stores are formed, enabling a rapid release of significant amounts of GDF-15 upon proteolytic cleavage (6). The pro-domain which remains in the ECM can be stained like a surface protein and has thus become the preferred target for immunohistochemical detection of GDF-15. Activation of GDF-15 is thought to be mainly mediated by furin (PCSK3) and other proprotein convertases of the subtilisin/kexin type, namely PCSK 5 and 6, which all cleave GDF-15 at the furine-like cleavage site RXXR (7). In placental cytotrophoblasts, processing and maturation can also occur via matrix metalloproteinase (MMP)-26 (8). Cleavage by membrane-type 1-matrix metalloproteinase (MT1-MMP), in contrast, abrogated autocrine effects in cancer cells (9). Interestingly, GDF-15 can also be detected in the nucleus, where it was reported to inhibit the Smad pathway by Smad complex disruption (10). Once fully processed, GDF-15 is released as a mature homodimer, with both molecules held together by a disulfide bond (11). Due to its low molecular weight (25 kD per dimer), mature GDF-15 is subject to renal clearance with a half-life of about 3 h in humans (12).

In healthy individuals, GDF-15 expression is by far most prominent in the placenta, followed by the prostate where both androgens and calcitriol (a Vitamin D metabolite) have been shown to regulate GDF-15 (13). Low levels of expression have been observed in the bladder, kidney, colon, stomach, liver, gall bladder, pancreas, and endometrium (11, 14). Cell types shown to express GDF-15 include cardiomyocytes, adipocytes, macrophages, endothelial and vascular smooth muscle cells both in healthy and diseased tissues [review by Tsai et al. (15)]. Being a stress-inducible cytokine, GDF-15 is (up-)regulated by several inflammatory or stress-related proteins such as interleukin (IL)-1ß, tumor necrosis factor (TNF)-α, interleukin-2, and macrophage colony-stimulating factor (MCSF)-1, suggesting a complex and tissue-specific regulation (16). Medication, cell stress and local interruption of blood supply can also induce GDF-15 during surgical procedures (17), which may contribute to the frequently elevated GDF-15 mRNA expression in surgical specimens. Still, in most tissues that can show inducible GDF-15 expression, physiological GDF-15 levels are low to absent in healthy individuals (14). Moreover, an all-male twin study revealed a significantly lower rate of survival for twins with elevated GDF-15 levels, with an odds ratio of 3.38 for death within 14 years (95%CI: 1.38-8.26) (18).

GDF-15 knock-out mice are viable and do not show any obvious phenotype after birth. A mild (≤20%) progressive loss of motor axons and rotarod motor skills are observed at 6 months suggesting a role for GDF-15 as a neurotrophic factor for motor and sensory neurons (19).

Elevated GDF-15 levels in cancer patients have been frequently reported. In a microarray-based study comparing 150 carcinomas from 10 anatomic sites of origin with 46 normal tissues derived from the corresponding tissues of tumor origin and other control non-transformed tissues, GDF-15 showed the highest level of tumor-associated (over)expression. Sera from patients with metastatic prostate, breast, and colorectal carcinomas validate this finding at protein level, which indicate that GDF-15 may be a biomarker for cancer (20). Cancer pathology specimens from the Protein Atlas database further confirmed elevated expression of GDF-15 in various types of cancer, most prominently in the prostate, urothelial, renal, melanoma, and colorectal cancers and, at relatively lower levels, in cervical, breast, endometrial, thyroid, and pancreatic cancers (21). Likewise, gene expression data from The Cancer Genome Atlas (TCGA) database show highest GDF-15 levels in prostate cancer and reveal also mRNA overexpression in breast, liver, colorectal, cervical, renal cell, hepato-, and cholangiocellular carcinomas (22, 23).

The numerous reported effects ascribed to GDF-15 in diverse malignancies (including intracranial brain tumors, melanoma, lung, gastrointestinal, pancreatic, colorectal, prostate, and breast epithelial cancers) include links between GDF-15 and tumorigenesis, disease progression, prognosis, clinical outcome and response to chemo- and radiotherapy (24, 25). Unfortunately, recombinant material sold by major distributors contained relevant amounts of TGF-β. This potential confounding factor has been described by several authors (15, 26, 27), and independently observed by Pierre Coulie (personal communication) and in our own unpublished experiments. Thus, use of contaminated GDF-15 has been a common problem, which has likely affected a substantial number of investigations in this field. Thus, the definitive role of GDF-15 in cancer and its possible role in immunotherapy remain to be elucidated.

GDF-15 was recently shown to signal through glial cell-derived neurotrophic factor (GDNF) family receptor α-like (GFRAL). GFRAL, which is distantly related to the TGF-β receptor family, is a neuronal high-affinity receptor for GDF-15 (28, 29). GFRAL is exclusively expressed in the human brain stem and is responsible for GDF-15 mediated anorexia (30, 31). However, anorexia and cachexia that should be caused by GDF-15-dependent activation of GFRAL are typically not observed during pregnancy, despite highly elevated GDF-15 serum levels. It also remains to be determined whether the effects of induced GDF-15 expression in pathological conditions such as metabolic diseases, tissue injury, inflammation, and cancer, all depend on GFRAL. Given that physiological effects of GDF-15 comprise manifold actions on cell types without detectable GFRAL expression, GFRAL-independent effects of GDF-15 appear plausible. This, in turn, leaves room for interactions between GDF-15 and yet unidentified receptor systems.

**Figure 1:**
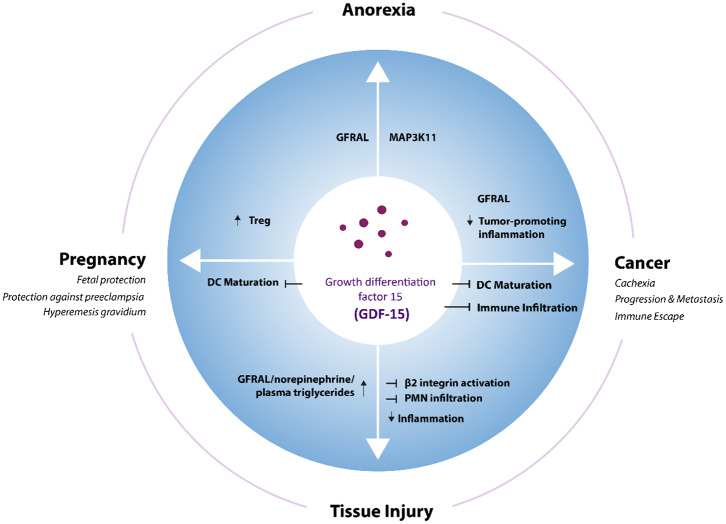
GDF-15 in different physiological and pathological contexts. GDF-15 stimulates or inhibits different cellular processes via GFRAL and potentially other still unknown receptors in various physiological and pathophysiological situations. DC, Dendritic cells; T_reg_, regulatory T cells.

Functionally, roles in appetite regulation, metabolism, cell and tissue survival, and immune tolerance have been described. This review aims to summarize the physiological and pathophysiological functions of GDF-15 (Figure 1), with a main focus on its role in cancer and cancer immunotherapy. Certain purity issues regarding commercially distributed GDF-15 reagents and their likely impact on many studies of GDF-15 mechanism of action (MOA) are also discussed. Finally, the emerging role of GDF-15 as a target for future cancer therapies is outlined.

## Role of GDF-15 in Metabolic Conditions

Anorexia and cachexia are metabolic syndromes characterized by loss of appetite, progressive weight loss, reduced adipose tissue, and skeletal muscle wasting (32). This condition is mediated by yet incompletely understood secreted factors from tissues or malignant cells. More than 80% of patients with advanced stage cancer experience anorexia and cachexia. As these metabolic disorders account for 20–30% of all deaths related to cancer, they represent a significant unmet medical need (32).

Several studies have shown a direct correlation between serum GDF-15 levels and anorexia/cachexia (29, 33). Appetite is centrally regulated by the hypothalamus region of the brain (34). GDF-15 has been shown to contribute to appetite loss in xenograft mouse models of prostate cancer; and a direct correlation between increased serum GDF-15 levels and cancer-associated anorexia has been observed in prostate cancer patients (35). In a subsequent study, the neurons responsible for GDF-15 dependent induction of anorexia and cachexia were localized to the *area postrema* and the nucleus of the solitary tract (36). Similar observations linking GDF-15 to anorexia/cachexia were made in various other conditions such as hepatocellular carcinoma (37). Using genetically engineered xenograft mouse models, the activation of mitogen-activated protein kinase kinase kinase 11 (MAP3K11) by GDF-15 was identified as the key trigger for weight loss in animal models of cancer-related cachexia (38). Weight loss could be reverted by neutralizing antibodies against GDF-15. Modulating GDF-15 in anorexia and cachexia, where GDF-15 is the prime regulator, might thus be therapeutically beneficial (38). The role of GDF-15 in weight regulation is further supported by the observation that GDF-15 transgenic mice are protected against obesity (39). GDF-15 deficient mice, in contrast, gain more weight when put on a high-fat diet (40). Finally, four independent research teams from four different pharmaceutical companies (Eli Lilly, Janssen, Merck, Novo Nordisk) managed to identify GFRAL (GDNF receptor alpha-like) as the brain stem receptor mediating the metabolic effects of GDF-15 (28–30, 41). Interestingly, GDF-15 production is also induced by metformin and, at least in mice, GDF-15 is responsible for the anti-obesity effects of this most commonly prescribed type 2 diabetes drug (42). Thus, GDF-15 and GFRAL are potential drug targets in the regulation of body weight and energy expenditure.

Conversely, researchers from the Novartis Institute for Biomedical Research found that anorexia and muscle loss, as complications in cancer, are mainly caused by increased levels of GDF-11, with GDF-15 being upregulated in response to supraphysiologic administration of GDF-11. Blockade of GDF-11 prevented both anorexia and muscle loss, whereas inhibition of GDF-15 was most effective against anorexia (43). A recent report on pharmacological GDF-15 administration to mice, which triggered conditioned taste aversion, also links GDF-15 more closely to anorexia than to cachexia and muscle wasting (33).

## GDF-15 as a Mediator of Immune Tolerance in Non-Cancer Conditions

GDF-15 has consistently been found to be induced in diseases involving immune homeostasis and surveillance and their regulation. Consequently, GDF-15 is implicated in physiological and pathological states where immune activation, immune surveillance and immune tolerance need to be finely balanced, because dysfunction and tissue damage are at stake.

### GDF-15 in Pregnancy

The highest GDF-15 expression is found in the placenta and the fetal membrane (11, 14). The hypothesis that GDF-15 plays a role in feto-maternal immunotolerance was formulated in 1997 (2). Subsequent studies showed that GDF-15 serum levels are increased in pregnant women at the onset of pregnancy and reach their highest concentration at the beginning of the third trimester (44). A retrospective study on sera collected during weeks 7–13 of pregnancy found comparatively lower GDF-15 serum levels in women who subsequently experienced miscarriages (45, 46). Likewise, the observation that GDF-15 levels are elevated to a lesser extent in women with preeclampsia (with the more profound reduction found in late-onset cases) suggests that GDF-15 is a potential biomarker for tracking pregnancy and pregnancy-related complications (47).

The constitutive production of GDF-15 in the prostate reaching the semen may also contribute to the success of pregnancy. While GDF-15 in seminal plasma does not affect the vitality of sperm cells, GDF-15 suppresses proliferation of peripheral blood mononuclear cells (PBMCs) and induces a regulatory phenotype in CD4^+^CD25^+^ cells via induction of FOXP3. Thus, GDF-15 may protect sperm cells from the maternal immune system (48). Further experimental support for the ability of GDF-15 to prevent allograft rejection comes from transplantation studies, where co-injection of GDF-15-overexpressing dendritic cells prolonged the average survival of C57BL/6-derived cardiac allografts in BALB/c mice from 14 to 77.5 days, and 4/6 grafts remained fully functional for over 100 days post-transplantation. GDF-15 thus has a potent tolerogenic function and may be involved in the protection of the semi-allogeneic fetus from immune-mediated rejection by the host immune system.

Interestingly, induction of anorexia and cachexia are rarely observed during pregnancy. Nevertheless, *hyperemesis gravidarum* (severe nausea and vomiting), which is reported in ~2% of pregnant women, is linked to polymorphisms in GDF-15 or in insulin-like growth factor-binding protein (IGFBP)7 (49). It remains to be determined whether the GDF-15 polymorphism affects the signaling function of GDF-15.

### GDF-15 in Metabolic Syndrome

Due to its metabolic function, GDF-15 induces a lean phenotype in transgenic mice (50). Besides shifting the balance between obesity and leanness, GDF-15 also acts on immune cells in adipose tissue. Of particular relevance for metabolic syndrome are effects on macrophages: Lean, non-inflamed adipose tissue is inhabited by anti-inflammatory M2-like macrophages (51) which cover their metabolic demands via oxidative phosphorylation (52, 53). In contrast, white adipose tissue of obese individuals is infiltrated by CD8^+^ effector T cells. These, in turn, promote recruitment and activation of predominantly glycolytic, pro-inflammatory M1 macrophages (54). An excess of M1 over M2 macrophages causes metabolic changes and increased TNF-α levels, leading to glucose intolerance and insulin insensitivity (55, 56).

In response to anti-inflammatory signals, GDF-15 is locally induced in white adipose tissue macrophages (57, 58). Induction of metabolic syndrome in GDF-15 knock-out mice revealed that IL-4, IL-13, or rosiglitazone-based treatments depend on the JAK/STAT6- or PPARγ-dependent upregulation of GDF-15 in macrophages. In an autocrine and paracrine loop, GDF-15 then activates oxidative phosphorylation in resident and recruited macrophages, thereby (re-)polarizing them toward an anti-inflammatory M2 phenotype (52, 57). GDF-15 deficient macrophages, in contrast, retain their M1 polarization even in the presence of anti-inflammatory cytokines. In line with its initial name *macrophage inhibitory cytokine*, GDF-15 thus limits pathological adipose tissue inflammation, reverts insulin resistance and ameliorates metabolic syndrome by metabolically modulating macrophage function. In humans, however, elevated GDF-15 represents a predictor for the future development of type 2 diabetes (59, 60) and, possibly, disease severity (16, 61). This still does not exclude a possible role for GDF-15 in delaying disease onset in patients at risk.

### GDF-15 in Tissue Injury and Inflammation

Being an inflammatory and stress-induced cytokine, expression of GDF-15 is often increased upon tissue injury. Two studies have reported an induction of GDF-15 in response to liver injury (62, 63). In the earlier study, GDF-15 expression was found to be rapidly and dramatically up-regulated following various surgical and chemical treatments that cause acute liver injury and regeneration (62). The latter study reported enhanced GDF-15 levels following carbon tetrachloride (CCl_4_)- or alcohol-mediated liver damage (63). Functionally, hepatic GDF-15 production ameliorates liver inflammation and fibrosis. Livers of GDF-15 knockout mice showed more severe fibrosis and increased infiltration of inflammatory CD4^+^ and CD8^+^ T cells, monocytes and neutrophils.

A protective function of GDF-15 on cardiac tissue was first demonstrated by a study using GDF-15 transgenic mouse models, where GDF-15 secreted by the myocardium acted as a protective and antihypertrophic factor (64). Exploring ischemia/reperfusion injury, GDF-15-deficient mice developed greater infarct sizes and displayed more apoptotic cardiomyocytes in the border zone, indicating that endogenous GDF-15 limits myocardial tissue damage *in vivo* (65). Induction of GDF-15 after myocardial infarction was shown to be essential for limiting the recruitment of polymorphonuclear leukocytes (PMNs), thereby permitting infarct healing without causing cardiac rupture (66). By demonstrating that GDF-15 limits the recruitment of infiltrating pro-inflammatory cells by interfering with chemokine signaling and β2-integrin/lymphocyte function-associated antigen 1 (LFA-1) activation, this study offered the first mechanistic explanation for anti-inflammatory effects of GDF-15.

Anti-inflammatory functions of GDF-15 are also apparent in a sepsis model, in which GDF-15 knockout mice mounted increased inflammatory responses to lipopolysaccharide (LPS), with increased expression of monocyte chemoattractant protein (MCP)-1, keratinocyte chemoattractant (KC)/mouse homolog of interleukin-8 (IL-8), IL-6, and TNF-α in both cardiac and renal tissues, finally leading to organ dysfunction. In wild-type and GDF-15 overexpressing mice, GDF-15 protected both cardiac and renal tissues from excessive inflammation, with LPS-induced sepsis not affecting the organs (67). Induction of GDF-15 during sepsis and its tissue-protective role were very recently confirmed, where the authors described GDF-15 as an “inflammation-induced central mediator of tissue tolerance” (68). Surprisingly, a GDF-15-dependent effect on tissue infiltration by pro-inflammatory immune cells in sepsis was not observed; rather, a different mechanism via activation of GFRAL inducing β-adrenergic signaling to stimulate the release of triglycerides from the liver was proposed. Cardio-protection is then achieved by maintaining triglyceride levels during acute inflammation. GDF-15 thus appears to promote metabolic adaptation to systemic inflammation (68). Interestingly, an earlier study from the same group has shown that switching to a fasting metabolism (which should be a consequence of GDF-15/GFRAL/RET-induced anorexia) can be life-saving in LPS- or Listeria-induced experimental sepsis (69).

By proposing that the central metabolic effects of GDF-15 are crucial for limiting inflammation-induced tissue damage, Luan et al. suggest an elegant link between the known functions of GDF-15 (68). Still, direct effects of GDF-15 on immune cells, as observed in previous studies (63, 66), would contribute toward a similar functional outcome. Irrespective of the underlying mechanism, there is consensus that GDF-15 protects the heart, liver and kidney after injury or stress, and regulates injury-mediated response in the lungs (70–72). Finally, an immunohistochemical analysis of benign atrophic lesions of the human prostate (where GDF-15 is constitutively expressed) revealed an inverse correlation between GDF-15 and infiltration by CD3^+^, CD4^+^, CD8^+^, CD68^+^, and iNOS^+^ leukocytes, whereas no correlation was observed with infiltration of arginase-positive (most likely myeloid-derived suppressor) cells (73). Thus, there is ample evidence that GDF-15 can limit inflammation-induced damage and help to preserve tissue integrity.

### Autoimmune Diseases

Autoimmune diseases result from often chronic aberrant immune responses against self-antigens with immune cells turning against host tissues. One of the most common autoimmune diseases is rheumatoid arthritis. Patients suffering from rheumatoid arthritis often display elevated GDF-15 levels, which correlate with symptoms such as erythrocyte sedimentation rate levels, morning stiffness, tender joint count, and carotid intima media thickness (74). For another autoimmune disease, Type 1 diabetes (T1D), elevated GDF-15 activity was described in beta cells (75), and GDF-15 was proposed as a biomarker for T1D (16). Functionally, GDF-15 was shown to protect pancreatic β-cells under inflammatory conditions and in non-obese diabetic mice. A possible protective effect against T1D was further corroborated by a reduced abundance of GDF-15 in *post mortem* islets from individuals with T1D (76). While these observations can be explained by the induction of GDF-15 under inflammatory conditions, a completely different outcome is observed in multiple sclerosis. In a well-characterized, longitudinally followed cohort of 48 patients with multiple sclerosis, GDF-15 was only elevated in a small subset of patients who were characterized by a stable course of disease with no relapse or further gadolinium-enhancing lesions over an average observational period of 5.9 years (77). Thus, in multiple sclerosis GDF-15 seems to be a biomarker for a stable course of disease rather than severity. A possible causal or functional relationship remains to be investigated.

Thus, GDF-15 seems to have a broad and diverse functional role in various conditions. Apart from its effects on appetite and body weight regulation, it protects the fetus by inhibiting T cells, protects other tissues against excessive inflammation, and is induced in many pathologies where GDF-15 function appears to be context-dependent.

## GDF-15 in Cancer

### GDF-15 as a Prognostic and Predictive Marker in Numerous (Solid) Cancer Entities

In a large-scale screening, GDF-15 was the most prominently overexpressed soluble factor across a large range of cancer types (20). GDF-15 was further proposed as a diagnostic biomarker in, e.g., colorectal (78, 79), ovarian (80), and early-stage lung cancer (79, 81). In fact, GDF-15 was found to be the most accurate marker at differentiating pancreatic adenocarcinoma from chronic pancreatitis (82). As an essential component of more complex marker panels, GDF-15 is a potential marker to aid in the discrimination between prostate cancer and benign hyperplasia (83–85). Correlations between GDF-15 and cancer progression have been described for colorectal (86, 87), gastric (88–91), hepatocellular carcinoma (92), non-small cell lung cancer (81), urothelial/renal cell (93), ovarian (80), melanoma (94), breast (20, 95), multiple myeloma (96, 97), and oral cancers (98). Findings from these studies indicate that GDF-15 is a promising prognostic marker for identifying cancer manifestation and progression.

GDF-15 is, however, more than a surrogate marker for tumor progression and tumor load. GDF-15 also remains highly predictive regarding clinical outcome in multi-variate analyses with other markers reflecting tumor burden (80, 81, 90, 94, 99). In colorectal cancer, a meta-analysis built upon eight individual studies concluded that higher GDF-15 expression is associated with worse overall survival, with a pooled hazard ratio (HR) of 2.09 [95% confidence interval (CI): 1.47–2.96] (78). In prostate cancer, GDF-15 serum levels independently predicted lower cancer-specific survival with an adjusted HR of 2.98 (95% CI: 1.82–4.68). Patients with high pretreatment GDF-15 levels even showed an 8-fold higher death rate than those with low GDF-15 (adjusted HR: 7.98; 95% CI: 1.73–36.86). Strikingly, a sequence variant in the GDF-15 gene that was associated with decreased GDF-15 serum levels (*P* = 0.002) was also associated with decreased mortality (*P* = 0.003), suggesting a disease-modifying influence of GDF-15 (100).

In patients with stage I and II non-small cell lung cancer, multivariate Cox regression survival analysis showed that high GDF-15 in serum was an independent risk factor for reduced overall survival (HR = 3.37, 95% CI: 1.09–10.42, *p* = 0.035) (81). In patients with tumor-free stage III or unresectable stage IV melanoma, there was such a significant link between elevated GDF-15 serum levels and poor survival that the clinically well-established marker LDH was no longer statistically significant in the multivariate analyses (94). In uveal melanoma, elevated GDF-15 correlated with the presence of metastases (*p* < 0.001) (101). In ovarian cancer, elevated GDF-15 was identified as an independent predictor for poor progression-free and overall survival, even after correction for FIGO stage and age (*p* = 0.01) (102, 103). GDF-15 was further reported to predict the failure of platinum-based chemotherapy and proposed as a diagnostic biomarker in ovarian cancer (80). In endometrial cancer, high plasma GDF-15 was, again, an independent predictor of poor disease-specific and short recurrence-free survival and was significantly associated with high tumor grade and lymph node metastasis (all *p* ≤ 0.001) (104).

In esophageal cancer, elevated GDF-15 was positively associated with tumor invasion (*p* = 0.030), lymph node metastasis (*P* = 0.007), and shorter relapse-free (*p* = 0.050) and tumor-specific survival (*p* = 0.005). Moreover, GDF-15 was the strongest predictor for outcome compared with other markers tested in the same patient cohort (105). Among patients with oral squamous cell carcinoma, those with elevated GDF-15 showed significantly shorter survival (*p* = 0.031) (98). In gastric cancer, increased levels of GDF-15 were associated with reduced progression-free and overall survival in univariate analyses; in multivariate analyses, high GDF-15 in combination with MMP7 and miR-200c was an independent predictor for death (*p* = 0.033) (90). In glioma, increased GDF-15 in cerebrospinal fluid correlated strongly with shorter survival (*p* = 0.007) (106). The association between high GDF-15 expression and poor survival of glioma patients was further confirmed following TCGA database interrogation (*p* = 0.017) (107). Similarly, in pancreatic cancer, an analysis based on 108 gene expression profiles from the TCGA database found a correlation between high GDF-15 expression and poor survival; however (possibly due to the generally dismal prognosis), this finding was not statistically significant (*p* = 0.105) (108).

In multiple myeloma where GDF-15 is produced by bone marrow stromal cells rather than by the tumor, increased GDF-15 serum levels were associated with a tumor-promoting microenvironment, as demonstrated by enhanced clonogenic growth of multiple myeloma cells and reduced progression-free survival (96). Apart from this study there is little evidence for the involvement of GDF-15 in hematological malignancies. In solid cancers, however, the epidemiological evidence for a correlation between elevated GDF-15 on protein level and poor survival is almost overwhelming (Table 1).

**Table 1:** Correlation between GDF-15 serum and/or tumor levels with clinical outcome in different cancer types.

**Indication**	**Patient cohort**	**Patient numbers**	**GDF-15 plasma/ serum assay**	**Assay matrix**	**Plasma/Serum threshold**	**GDF-15 expression tumor (IHC or RNA)**	**Correlation with clinical outcome and disease markers**	**References**
Breast cancer (BC)	Gustave Roussy Cancer Center, Villejuif, France	605 BC patients	Not applicable	Not applicable	Not determined	GDF-15 IHC of 605 BC patients	Tumor GDF-15 expression correlates with ER-negative and HER2-positive status in patients with breast cancer	(109)
Colorectal cancer (CRC)	Nurses' Health Study (NHS), Health Professionals Follow-up Study (HPFS), USA	616 CRC patients from 2 independent patient cohorts	GDF-15 ELISA (R&D Systems)	Plasma	1,060 pg/mL	Not applicable	Plasma GDF-15 above threshold correlates with shorter OS	(87)
	Zhejiang University Sir Run Run Shaw Hospital, China	138 CRC patients	GDF-15 ELISA (not defined)	Serum	1,150 pg/mL	Not applicable	Serum GDF-15 above threshold correlates with shorter OS	(110)
	Charles University and General University Hospital, Czech Republic	97 metastatic CRC patients	GDF-15 ELISA (Biovendor-Laboratorni medicina)	Serum	7,000 pg/mL	Not applicable	Serum levels of GDF-15 were significantly higher in patients with colorectal cancer compared to healthy controls. GDF-15 correlates with shorter OS.	(111)
	Nurses' Health Study (NHS), USA	757 CRC patients	GDF-15 ELISA (R&D Systems)	Plasma	Not determined	Not applicable	Elevated levels of plasma GDF-15 were associated with higher risk of advanced colorectal cancer	(112)
	The Johns Hopkins Hospital, USA St. Vincent's Hospital, Australia	525 CRC patients from 2 independent patient cohorts	GDF-15 ELISA (in-house)	Serum	1,150 pg/mL	Not applicable	Serum levels of GDF-15 were significantly higher in patients with colorectal cancer compared to healthy controls. Serum GDF-15 above threshold correlates with disease progression and shorter OS	(113)
	Vasteras Hospital, Sweden	320 CRC patients	GDF-15 SP-PLA	Plasma (60 patients)	1,150 pg/mL	GDF-15 IHC of 274 CRC patients	Moderate or high tumor GDF-15 intensity correlates an increased risk of recurrence and with shorter OS	(114)
	Chinese Academy of Medical Sciences (CICAMS),	473 CRC patients	GDF-15 ELISA (in-house)	Serum	1,000 pg/mL	Not applicable	Serum GDF-15 is significantly higher in patients with stage IV CRC compared to stage I-III and healthy individuals. Patients with liver metastasis have elevated serum levels of GDF-15. Disease recurrence is associated with increase of serum GDF-15 levels. Patients with higher serum GDF-15 had a trend to poorer tumor-specific survival	(79)
Endometrial Cancer	Oslo University Hospital & Haukeland University Hospital, Norway	510 endometrial cancer patients	Immunoradiometric sandwich assay using a polyclonal, affinity chromatography– purified goat anti-human GDF-15 IgG antibody (R&D Systems)	Plasma	Upper tertile of GDF-15 level (median = 1,077 pg(mL)	Not applicable	High plasma GDF-15 is associated with metastatic disease. GDF-15 above threshold correlates with shorter PFS and OS	(104)
	Haukeland University Hospital, Norway	235 endometrial cancer patients	GDF-15 ELISA (R&D Systems)	Plasma	Upper tertile of GDF-15 level (median = 1,780 pg/mL (range 518–9,475 pg/mL)	Not applicable	High plasma level GDF-15 independently predicts recurrent disease and lymph node metastases. GDF-15 above threshold correlates with shorter PFS and OS	(115)
Esophageal cancer	Chinese Academy of Medical Sciences (CICAMS), China	286 ESCC patients	GDF-15 ELISA (in-house)	Serum	1,000 pg/mL	GDF-15 IHC of 40 Patients	Serum GDF15 decreased after surgical removal and increased at relapse. GDF-15 above threshold correlates with shorter relapse-free survival and tumor-specific survival	(105)
Gastric cancer	Dankook University College of Medicine, Korea	80 gastric cancer patients	GDF-15 ELISA (R&D Systems)	Serum (80 patients)	Not determined	41 patients	Serum GDF-15 and tumor GDF-15 (protein and mRNA) expression is higher in patients with gastric cancer. Tumor GDF15 correlates with differentiation stage	(89)
Gastric cancer	Peking University Cancer Hospital, China	384 gastric cancer patients	GDF-15 ELISA (in-house)	Serum (217 patients)	1,120 pg/mL	Not applicable	Serum GDF-15 levels above threshold before chemotherapy and increased GDF-15 levels during chemotherapy correlate with shorter OS	(116)
	La Coruña Biomedical Research Institute (INIBIC), Spain	52 gastric cancer patients	GDF-15 ELISA (R&D Systems)	Serum	493 pg/mL	Not applicable	Serum GDF-15 above threshold correlates with shorter PFS and OS	(90)
Glioblastoma	Center Hospitalier Universitaire Vaudois, Lausanne, Switzerland	33 patients with glioblastoma	GDF-15 ELISA (in-house)	Plasma Cerebrospinal fluid (CSF)	156 pg/mL	Not applicable	GDF-15 (CSF) above threshold correlates with shorter OS	(106)
	TCGA database (http://cancergenome.nih.gov),	540 glioblastoma patients (TCGA)	Not applicable	Not applicable	Not determined	GDF-15 tumor mRNA expression obtained from TCGA	Low GDF-15 tumor mRNA expression correlate with better outcome in glioblastoma	(107)
Head and neck cancer (HNSCC)	Johannes Gutenberg-University Mainz, Germany	64 OSCC patients	GDF-15 ELISA (R&D Systems)	Serum	875 pg/ml	Not applicable	Serum GDF-15 above threshold correlates with shorter OS and tumor load	(98)
	Ninth People's Hospital, Shanghai Jiao Tong University School of Medicine, China	256 stage III and IV OSCC patients	Not applicable	Not applicable	Not determined	GDF-15 IHC of 256 patients	Elevated tumor tissue expression of GDF-15 correlates with shorter PFS and OS. Docetaxel, cisplatin and 5-fluorouracil (TPF) induction chemotherapy is beneficial for patients with elevated tumor GDF-15 expression	
	Ninth People's Hospital, Shanghai Jiao Tong University School of Medicine, China	60 OSCC patients	GDF-15 ELISA (R&D Systems)	Serum	364 pg/mL	Not applicable	GDF-15 above threshold did not significantly correlate with shorter OS	(117)
Melanoma	University Hospital Tubingen, Germany	761 stage III/IV melanoma patients	GDF-15 ELISA (R&D Systems)	Serum	1,500 pg/mL	Not applicable	Elevated GDF-15 correlates with shorter OAS	(94)
Melanoma	Oslo University Hospital The Norwegian Radium Hospital, Norway	69 patients with unresectable or metastatic malignant melanoma were treated with ipilimumab (Phase IV study (NCT0268196/EudraCT2013-002408-15)	GDF-15 ELISA (R&D Systems)	Serum (56 patients)	517 pg/mL	Not applicable	In univariable analysis baseline GDF-15 above threshold correlated with shorter survival. The association between survival and GDF-15 was markedly attenuated by multivariable adjustment and was no longer significantly associated with death under subsequent ipilimumab treatment	(118)
	Pennsylvania State University, USA	29 melanoma patients	GDF-15 ELISA (R&D Systems)	Serum (10 patients)	Not determined	22 patients	Primary melanoma biopsies expressing low levels of GDF-15. Metastatic melanoma patients having elevated GDF-15 expression in tumors also had high levels of GDF-15 in serum	(119)
	Queensland Institute of Medical Research and Princess Alexandra Hospital	22 primary melanoma and 16 metastatic melanoma patients	Not applicable	Not applicable	Not determined	38 patients	Elevated GDF-15 tissue expression, detected by IHC, was significantly associated with metastatic melanoma and not primary melanoma	(120)
Hepato-cellular Carcinoma (HCC)	Chinese Academy of Medical Sciences (CICAMS), China	223 HCC patients	GDF-15 ELISA (R&D Systems)	Serum	2.463 ng/mL	GDF-15 IHC of 20 HCC patients	Tumor GDF15 protein expression in HCC was significantly higher than that in the corresponding adjacent paracarcinomatous tissue and normal liver. Serum GDF15 level is elevated in patients with HCC	(92)
Multiple myeloma	Franccophone du Myelome treatment trials, France	131 multiple myeloma patients	GDF-15 ELISA (R&D Systems)	Plasma	500 pg/mL		GDF-15 correlates with shorter event-free survival and OS and β2-microglobulin level and disease stage	(121)
	University Hospital Malmo, Sweden	138 multiple myeloma patients	GDF-15 MILLIPLEX MAP Human Panel (Millipore)	Serum	1,008 pg/mL		GDF-15 above threshold correlates with shorter OS	(97)
	The Johns Hopkins Hospital, USA	15 multiple myeloma patients	GDF-15 ELISA (R&D Systems)	Serum	Not determined	Not applicable	Increased serum GDF-15 levels correlate with shorter OS	(96)
Non-small cell lung cancer (NSCLC)	Nanjing Medical University Jiangsu, China	46 stage I/II and 20 stage III/IV NSCLC patients	Not applicable	Not determined	Not determined	GDF15 mRNA by qRT-PCR of 66 NSCLC patients	Downregulated GDF15 mRNA in NSCLC tissues is correlated with poor clinical outcomes in NSCLC	(122)
	Cancer Institute and Hospital Beijing, China	152 stage I and II NCSCL patients	GDF-15 ELISA (in-house)	Serum	1,465 pg/mL		Serum GDF-15 above threshold correlates with shorter OS	(81)
Esophageal adeno-carcinoma (OAC)	St. Vincent's Hospital, Australia & PROBE-NET study, Australia	138 OAC patients	GDF-15 ELISA (in-house)	Plasma	1,140 pg/mL	GDF15 mRNA by MT-PCR of 138 OAC patients	GDF15 mRNA tissue expression is higher in esophageal adenocarcinoma compared to healthy individuals. Plasma GDF-15 above threshold correlates with shorter OS in OAC	(123)
Pancreatic cancer	Arthur G. James Comprehensive Cancer Center, USA	27 PDAC patients, 183 PDAC patients from the TCGA database.	GDF-15 ELISA (R&D Systems)	Serum	Not determined	Not applicable	GDF15 mRNA expression shows trend for shorter OS	(108)
Ovarian cancer	Xijing Hospital, China,	145 EOC patients	GDF-15 ELISA (USCNLIFE)	Serum (120 patients)	748 pg/mL	145 patients	High GDF-15 expression in EOC tissue showed shorter PFS and OS. Serum GDF-15 levels where higher in EOC patients resistant to first-line chemotherapy	(103)
	National Cancer Center/Cancer Hospital, China	122 EOC patients	GDF-15 ELISA (CICAMS)	Serum	960 pg/mL	Not applicable	Serum GDF-15 above threshold correlates with PFS and platinum-refractory disease	(80)
	Oslo University Hospital, Ulleval, Norway	312 ovarian cancer patients	Immunoradiometric sandwich assay)	Plasma	1,242 pg/mL	Not applicable	Plasma GDF-15 above threshold correlates with shorter OS	(102)
Ovarian Cancer	Federal University of Triângulo Mineiro, Uberaba, Brazil	38 ovarian cancer patients	GDF-15 ELISA (Aviscera Bioscience Inc.)	Serum	Not determined	59 patients	Serum GDF-15 levels were higher in the patients with malignant neoplasms than in the patients with benign tumors, yet the difference was not statistically significant. GDF-15 immunostaining was significantly more frequent in the stroma of the malignant tumors than in the stroma of the benign tumors	(124)
Prostate cancer	Cancer Prostate, Sweden	1,442 prostate cancer patients	GDF-15 ELISA (in-house)	Serum	1,466 pg/mL	Not applicable	Serum GDF-15 above threshold correlates with shorter OS	(100)
	King George's Medical University, Lucknow, Center of Biomedical Research, SGPGIMS Campus Lucknow, India	85 prostate cancer patients	GDF-15 ELISA (in-house)	Serum	Not determined	Not applicable	GDF-15 correlates with PSA and Gleason score	(85)
	University Hospital Munster, Germany	38 prostate cancer patients	GDF-15 ELISA (R&D Systems)	Serum	1,300 pg/mL	Not applicable	Serum GDF-15 levels were higher in metastatic than in non-metastatic patients. Serum GDF-15 above threshold correlates with shorter OS	(125)
Renal Cell Cancer (RCC)	University Hospital Munster, Germany	94 RCC patients	GDF-15 ELISA (R&D Systems)	Serum	1,200 pg/mL	Not applicable	Serum GDF-15 is linked to metastases and relapse. Serum GDF-15 above threshold correlates with shorter OS	(93)
Urothelial carcinoma (UUTUC)	University Hospital Munster, Germany	38 UUTUC patients	GDF-15 ELISA (R&D Systems)	Serum	1,200 pg/mL	Not applicable	Serum GDF-15 is linked to metastases and relapse. Serum GDF-15 above threshold correlates with shorter OS	(93)
Uveal melanoma (UM)	University Hospital Tubingen, Germany	188 uveal melanoma patients	GDF-15 ELISA (R&D Systems)	Serum	Not determined	GDF-15 IHC of 44 UM patients	Patients with clinically detectable metastases had significantly higher GDF-15 serum levels compared to those without clinically detectable metastases as well as to healthy individuals	(101)
	The Johns Hopkins Hospital, USA	48 uveal melanoma patients + 36 healthy controls	GDF-15 Bio-Plex (BioRad)	Serum	Not determined	Not applicable	GDF-15 + MIA proposed to discriminate between patients with metastatic uveal melanoma and disease-free patients [AUC = 0.85 (0.68–1.00), n.s.]	(126)
Solid vs. non-solid treatment-naïve malignancies	Vienna General Hospital	555 treatment-naive cancer patients (breast cancer, lung cancer, gastrointestinal cancer and hematological cancers)	GDF-15 ELISA (R&D Systems)	Serum	Tertiles of GDF-15 level	Not applicable	GDF-15 is significantly associated with outcome for solid tumors as breast cancer, lung cancer or gastrointestinal cancer; however, no association with outcome could be shown for hematological cancers as myelodysplastic or myeloproliferative diseases	(127)

A disease-modifying effect of GDF-15 is further supported by findings that a polymorphism in the GDF15 gene (H6D) affects both tumor risk and prognosis in colorectal (113, 128) and in prostate cancer (HR: 0.83 or 0.85 from two different studies) (129, 130). Interestingly, the H6D genotype is associated with lower tumor risk and correlates with more aggressive growth and an increased risk of death (HR: 1.72; 95% CI: 1.06-2.78; *p* = 0.03), once prostate cancer has developed (130).

As a potential biomarker for immunotherapy, GDF-15 was included in a study exploring possible predictors for resistance to anti-CTLA-4 (Ipilimumab) therapy. In this analysis, GDF-15, endostatin, osteoprotegerin, C-reactive protein, pulmonary and activation-regulated chemokine and galectin-3 binding-protein that were persistently higher in non-survivors. Statistically, however, effects were significant only prior to multivariable adjustment (*p* = 0.01 for GDF-15) (118). However, as this study was only based on serum samples from only 56 melanoma patients, a possible correlation remains to be further explored in a larger cohort.

### GDF-15 in Cancer-Induced Anorexia/Cachexia

A prevalent and often fatal complication in patients affected with cancer is anorexia and cachexia syndrome (32). Based on the initial observation that tumor-derived GDF-15 induces this condition (35), on the localization of the underlying signaling mechanism to the brainstem (131), and on the identification of the brainstem-restricted GDF-15 receptor GFRAL and its signaling pathway via RET (28–30, 41), GDF-15 and GFRAL have become major targets for appetite and weight regulation. The subsequent finding that GDF-15 is primarily responsible for the loss of appetite, whereas loss of muscle mass depends more on GDF-11 (43), may, however, limit the usefulness of anti-GDF-15 (mono)therapy in cancer patients with muscle wasting syndrome.

### GDF-15 in Tumor Immune Evasion

The ability of tumor cells to evade immune surveillance (132, 133) is now considered a hallmark of cancer (134). The immunological contexture within the tumor microenvironment has also been recognized as a major predictor for survival (135, 136). This immune effect is reflected in the tremendous predictive power of Immunoscore (137, 138) which defines the immune “fitness” of a given tumor host interaction-based on the localization of CD8 T lymphocytes in the invasive front and the center of the tumor. Moreover, the presence of tumor-adjacent organized lymphoid aggregates and the type of inflammatory context are also strongly linked to outcome (139).

**Figure 2:**
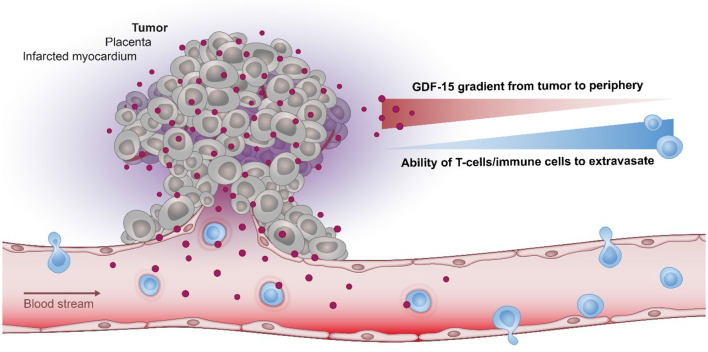
The role of GDF-15 in immune modulation. In various pathological conditions, GDF-15 correlates inversely with the ability of T cell to infiltrate the tumor, placenta, or the infarcted myocardium. As the most prominent physiological expression of GDF-15 is found in the placenta it may have evolved to protect the (semi-allogeneic) fetus by establishing a protective barrier at the placenta-fetal junction, thus shielding the fetus against maternal T cells.

Based on its ability to shield tissues against inflammation (Figure 2), GDF-15 may thus be expected to change the immune contexture within a tumor. In line with this hypothesis, an observational study reported an inverse correlation between elevated GDF-15 expression and the presence of a Crohn's-like type of mononuclear infiltrate (113). Interestingly, this negative correlation was not found in a subgroup of patients with an allelic variation in GDF-15, indicating that immune exclusion in GDF-15 overexpressing tumors might be linked to functional properties of the most common GDF-15 allele. A further study in transplantable glioblastoma revealed that shRNA-mediated downregulation of GDF-15 increased T cell infiltration into tumors, improved immune responses and prolonged survival (140).

GDF-15 was further identified in an unbiased screening for tumor-derived factors that suppress dendritic cell function. Functional assays showed GDF-15 to be a potent suppressor of dendritic cell maturation that inhibits expression of co-stimulatory and major histocompatibility complex (MHC) class II molecules, reduces IL-12 levels and elevates TGF-β1 secretion. Thus, GDF-15 may promote immune escape of tumor cells by inhibiting T cell stimulation and cytotoxic T lymphocyte activation by dendritic cells (99). Similar to macrophages in white adipose tissue (52, 57) also macrophages in the tumor microenvironment adopt a tolerogenic/immunosuppressive phenotype when exposed to GDF-15. Depletion of tumor-derived GDF-15 in an orthotopic pancreatic cancer model and in Ras-driven tumor xenografts hence restored the immune surveillance function of tumor-infiltrating macrophages, resulting in improved tumor control (108). Thus, a role for GDF-15 in tumor immune escape is supported by many studies. Still, there is no consensus regarding the underlying mechanism(s).

### Mechanisms of Action

With such a broad spectrum of functions across tissues and cellular functions (Figure 1), it is crucial to identify the mode of action of GDF-15, its interacting receptors, and their downstream signaling pathways. The recognition of GDF-15 as an important regulator of body weight (35) has initiated a search to identify its potential partnering receptor and the resulting signaling pathways. From earlier studies, it was postulated that the signaling pathway of GDF-15 would be through SMAD2/3 interacting with the receptor TGFβRII (64, 141, 142). In the four independent studies which identified GFRAL to be the receptor responsible for the metabolic effects of GDF-15, binding of GDF-15 to members of the TGF-β receptor superfamily was thus carefully evaluated (28–30). However, binding of ^125^I-labeled GDF-15 (30 pM) to COS7 cells transfected with combinations of type I and type II TGFβ family receptors (30); flow cytometry-based binding assays using biotinylated GDF-15 on HEK cells transfected with GFRAL and all 139 other molecules related to the TGF-β receptor superfamily (41); and cell-based PathHunter Dimerization assays on more than 20 potential TGF-β-family receptor pairs (29) all failed to show any binding of GDF-15 to a TGF-β receptor. Thus, for now the GDNF receptor family member GFRAL remains the only validated GDF-15 receptor (143). Like other members of the GDNF receptor family, GFRAL interacts with its co-receptor RET (most efficiently with RET51) and signals via the extracellular-signal related kinase (ERK) and AKT/protein kinase B pathways, without activating canonical TGF-β signaling pathways.

Very importantly, several authors have reported that commercially available GDF-15 preparations are often contaminated with variable bioactive levels of TGF-β1 ((15, 26, 27) and others [Pierre Coulie, personal communication]). Signal transduction through SMAD2/3 or via TGFβRII interaction may thus be artifacts caused by contaminating TGF-β1 rather than by GDF-15 itself (7, 52, 144). Therefore, studies reporting TGF-β related effects induced by recombinant human GDF-15 need to be regarded with great caution. Consequently, this review largely focusses on findings from genetic *in vivo* models and on *ex vivo* analyses from human samples. Still, there remains substantial evidence for direct effects of GDF-15 on leukocytes. Given that GFRAL expression is restricted to brainstem neurons, binding of GDF-15 to GFRAL cannot account for these immunomodulatory effects of GDF-15. Experiments comparing immunological alterations in GDF-15 knock-out mice vs. GFRAL knock-out mice would be a straightforward approach to clarify whether GFRAL-independent effects of GDF-15 exist.

Interestingly, a GFRAL- and CNS-dependent metabolic loop has been identified by which GDF-15 induces disease tolerance largely independent of pathogen control or the magnitude of inflammatory response (68). Instead, the authors propose that GDF-15 acts as an inflammation-associated hormone, coordinating tolerance to inflammatory damage through regulation of hepatic triglyceride metabolism. While this links the tissue-protective function of GDF-15 to its well-understood metabolic effects, the proposed loop places any effect of GDF-15 downstream of a systemic metabolic shift. This proposed mechanism thus cannot account for local or fast-acting effects of GDF-15. Similarly, the role of GDF-15 in shaping the tumor microenvironment at very early tumor stages (108) is unlikely to be attributable to a CNS-mediated systemic change of metabolism. In consideration of the substantial amount of data describing anti-inflammatory functions of GDF-15 (63, 66), the inability of GDF-15 to modulate the extent of inflammation in the chosen sepsis model is also surprising. Possibly, the inflammatory burst in the chosen model was strong enough to overrule local immune-inhibitory effects of GDF-15, thus revealing a secondary systemic level of GDF-15 dependent tissue protection.

### Signaling of GDF-15 in Cancer Cells

Given the very limited tissue distribution of GFRAL (28–30, 41), direct effects of GDF-15 on cancer or immune cells are likely to be mediated via GFRAL-independent signaling pathways. With regard to tumor cells, GDF-15 has been implicated in suppression or stimulation of tumor cell apoptosis, in early tumorigenesis, in epithelial-to-mesenchymal transition (EMT), in stemness, chemo-resistance, angiogenesis invasion, and metastasis [review by Modi et al. (25)]. Signaling mechanisms ascribed to GDF-15 in various carcinomas do not only involve Smad 2/3 signaling, which could also be attributable to contaminating TGF-β in preparations of GDF-15, but also IGFR1, PI3K, Akt, ERK1/2, β-catenin, p38, MAPK, Smad1/5/8, NF-κB, reactive oxygen species, mTOR, FAK–RhoA, EGFR, ErbB2, and c-myc signaling (among others). An understanding of which pathway is activated in which cell type or situation is, unfortunately, completely lacking.

In breast cancer, where GDF-15 has been linked to metastasis and to resistance toward trastuzumab, the following signaling pathways were suggested for GDF-15: Signaling via Smad 1/5/8 leading to up-regulation of hepcidin (145); induction of IGF1R-FoxM1 signaling leading to activation of Snail and Slug, epithelial-to-mesenchymal transition and MMP2/9-mediated cellular invasion (109); triggering of HER2-independent, TGFR- and Src-dependent phosphorylation of the HER2-Akt-Erk1/2 axis (146, 147); activation of p38 MAPK (148) and of JNK mitogen-activated protein kinases (MAPKs) (149) resulting in enhanced invasion of HER2-positive cancer cells. In contrast, another study found inhibition of invasion and of metastasis via activation of the Yes associated protein (YAP) leading to (YAP)-dependent transcriptional repression (150).

In cervical cancer cell lines, GDF-15 was found to act through a complex with ErbB2, thereby stimulating PI3K, AKT1, Erk1/2, and Ras-GTP signaling. Enhanced cell proliferation and up-regulation of cdc25A, CDK2, CDK4, CyclinD1, CyclinE1, as well as down-regulation of p21 were found to be FOXO1- and c-Myc-dependent (151). In lung cancer cells, GDF-15 was described to inhibit rather than activate the p38-MAPK (152) and the PI3K-Akt-PKB and ERK1/2 pathways (153), resulting in enhanced apoptosis. In colorectal cancer, where clinical data have established a strong link between GDF-15 expression and poor survival, GDF-15 is reported to induce EMT-related factors (154) and to enhance metastasis (78). Another study has, however, described GDF-15 dependent induction of apoptosis in colorectal cancer cells and suggested GDF-15 to act as a tumor suppressor (155).

In prostate cancer, where GDF-15 expression was also found in tumor-associated fibroblasts, GDF-15 was positively associated with tumor cell proliferation, cancer progression and anchorage-independent growth (156–158). Proposed mechanisms include activation of FAK–RhoA signaling leading to actin rearrangement and enhanced cell motility, invasion and metastasis (159) and dysregulation of maspin, matriptase, and IL-6 (157). In ErbB2-overexpressing gastric cancer cells, GDF-15 was found capable of transactivating EGFR family tyrosine kinases leading to Akt and ERK-1/2 phosphorylation and enhanced invasion. Further, ERK-dependent induction of urokinase-type plasminogen activator and urokinase type plasminogen activator receptor was reported, again leading to enhanced metastasis (146). Others, again, observed induction of apoptosis by GDF-15 in gastric cancer cells (160). In hepatocellular carcinoma, GDF-15 was found to enhance cell viability, invasion, metastasis (79), and hepatitis C virus (HCV) replication (161). Findings for hepatocellular carcinoma also include pro-apoptotic/anti-tumor effects of GDF-15, whereby GDF-15 has been linked to inhibition of Bcl-2 and Bcl-xL, upregulation of Bax, activation of pro-caspase 3, 8, and 9, and PARP (poly (ADP-ribose) polymerase) cleavage (162–164). In stem cells derived from hepatocellular cancer, GDF-15 induced AKT/GSK-3β/β-catenin signaling, which would, again, be tumor-promoting (165).

In MyD88-positive type I epithelial ovarian cancer cells, GDF-15 induced NF-κB signaling *in vitro*, thereby up-regulating stemness markers (OCT-4, SOX-2) and chemokine expression (CXCL-1, IL-8, and MCP-1) (166). In other studies on ovarian cancer cells, GDF-15 was found to signal via PI3K/mTOR, MAP kinases, phosphorylation of p38, Akt, and 4EBP1 to promote proliferation, anchorage-independent growth, invasion and up-regulation of matrix metalloproteinases MMP2/9 and vascular endothelial growth factor (VEGF) (167). GDF-15 has also been found to functionally contribute to platinum resistance (168). In immortalized oral mucosal and oral squamous cell carcinoma (OSCC) cell lines, GDF-15 overexpression also resulted in increased phosphorylation of Akt and ERK1/2 and significant induction of cell proliferation, migration, invasion, and colony formation (169). In addition, GDF-15 increased radio-resistance of OSCC (170), most likely by reducing intracellular reactive oxygen species (171). Anti-apoptotic activity of GDF-15 was shown *in vitro*, where overexpression of GDF-15 reduced caspase 3/7 activity and knockdown of GDF-15 increased the level of cleaved PARP and BAX (98). Paradoxically, GDF-15 was also reported to enhance apoptotic effects of tolfenamic acid (TA) via increased caspase-3 cleavage and cleaved poly(ADP-ribose) polymerase (PARP) in HNC cells (172).

In melanoma where GDF-15 expression is induced by oncogenic V600EB-Raf (119) and by microphthalmia-associated transcription factor (MITF) (120), GDF-15 may be involved in angiogenesis (119). Knockdown experiments in pancreatic cancer cells showed GDF-15 to be an effector of Twist-mediated changes, including enhanced invasion and chemoresistance (173). In glioma cells, RNAi-mediated GDF-15 depletion inhibited tumor cell proliferation and enhanced immunogenicity, immune infiltration, and survival in a syngeneic mouse model (140).

Thus, *in vitro* and *in vivo* studies suggest that the effects of GDF-15 on cancer cells are manifold, even when no GFRAL-expressing neuronal cells are present. As opposed to the evident epidemiological data linking GDF-15 with poor survival, studies exploring autocrine effects in tumor cells have, however, reached no consensus as to whether GDF-15 acts as a tumor promoter or suppressor. An understanding of which of the possible signaling pathways is likely to be activated by GDF-15 in a specific situation is also lacking and difficult to predict, since key pieces of the puzzle are still missing. It is unclear whether these diverse outcomes ensue from cell-intrinsic signaling, which is instigated by GDF-15's action on tumor cells. Alternatively, the highly context-dependent findings might best be explained by GDF-15's action on the tissue context of the tumor cells, i.e., the tumor microenvironment.

### Signaling of GDF-15 in the Tumor Microenvironment

The tumor microenvironment is typically composed of non-malignant cells, including fibroblasts, vascular cells, lymphatic vessels, and immune cells of lymphoid or myeloid origin. It provides a niche for tumor cells, protecting them against immunosurveillance and enabling them to retain a dedifferentiated, stem cell-like state. The various reports on GDF-15 promoting stemness in cancer cells (96, 165, 166, 171) might thus be explained by GDF-15 shaping the tumor microenvironment. Moreover, the diagnostically most essential cells in the tumor microenvironment are immune cells. It is now clear that changes in the immune contexture of a tumor have a significant impact on the biological behavior of cancers and on patient survival (136). Context-dependent strong effects of GDF-15 could thus be explained by GDF-15 inducing changes in the immune contexture. Thus, the GFRAL- and hepatic triglyceride-dependent tissue-protective effect (68) could also support the survival of neoplastic tissue when attacked by an immune response and thereby change the delicate balance between tumor and immune cells.

As outlined above, there is also ample evidence for direct effects of GDF-15 on immune cells. These effects include the well-documented function of GDF-15 as immune cell repellent (63, 66, 73, 140) (Figure 2), which can be explained by GDF-15 inhibiting the conformational activation of the integrin LFA-1 (lymphocyte function-associated antigen 1, αLβ2-integrin, CD11a/CD18). However, while the initial observation that GDF-15 interferes with LFA-1 dependent recruitment of inflammatory cells was convincingly shown *in vivo* (66), a subsequent study suggesting that these effects would be mediated through the ALK-5/TGF-βRII heterodimer (142) may have been affected by use of contaminated reagents (27, 29, 30, 41). Still, the mechanistic data generated with knock-out and heterozygous mice remain valid. Furthermore, the key role of LFA-1 at the immunological synapse (174) would predict that GDF-15's role is not restricted to immune cell recruitment (63, 66, 73, 113, 140). Inhibition of LFA-1 by GDF-15 would also impair the priming of antigen-specific T cells by dendritic cells. Such functional outcomes were independently observed in other studies (99, 175). The latter study also found that a knock-out of GDF-15 in immature dendritic cells promotes their maturation and enhances their immune stimulatory potential. Transgenic expression of GDF-15 in dendritic cells, in contrast, induces immune-inhibitory molecules in dendritic cells which then enhance T cell exhaustion and promote the generation of regulatory T cells. Mechanistically, the observed GDF-15 mediated induction of a tolerogenic dendritic cell phenotype was found to depend on TGF-β receptors I and II (rather than on GFRAL), on repression of malate-1 circular RNA expression, on inhibition of NF-κB signaling, and on the induction of indoleamine-2,3-dioxygenase (IDO1) in dendritic cells (175). Effects of GDF-15 on LFA-1 were not investigated and possible problems resulting from the use of recombinant human GDF-15 (which may have contained TGF-β) were not discussed. Still, the *in vivo* findings appear convincing and impressive.

Another study based on pancreatic cancer models indicated that tumor cells use GDF-15 during the early stages of tumorigenesis to evade macrophage-mediated immune surveillance. Mechanistically, tumor-derived GDF-15 was reported to suppress proapoptotic macrophage activity by inhibiting NF-κB signaling via TGF-beta-activated kinase (TAK1), thereby blocking the synthesis of TNF-α and nitric oxide (NO) production (108). Thus, different studies suggest different mechanisms to explain the effects of GDF-15 on immune cells. There is, however, a consensus that GDF-15 inhibits immune responses at different levels. Further, there is evidence for inhibitory effects on immune cell recruitment, on the function of antigen-presenting cells and on macrophage polarization. Thus, the different reports appear complementary rather than contradictory.

Perceiving GDF-15 as an anti-inflammatory molecule in cancer may then also explain paradoxical findings of GDF-15 overexpression on prostate cancer development and spread. In the TRAMP transgenic model of spontaneous prostate cancer, transgene-mediated overexpression of GDF-15 reduced the growth of the primary tumor (176). As tumor-promoting inflammation (which has been declared a hallmark of cancer since 2011) (134) critically contributes to spontaneous tumor development in this model (177, 178), suppression of tumor growth by GDF-15 may be explained by its anti-inflammatory effects. Metastasis is, however, limited by immune surveillance in this (179) and in many other tumor models (179–183). Since GDF-15 transgenic mice developed more lung metastases and distant organ metastases, metastasis formation does not seem to depend on the size of the primary tumor. Instead, metastasis may be facilitated by effects of GDF-15 on immunosurveillance (176). Likewise in patients, where larger-sized prostate tumors can be resected, while metastasis is a deadly threat, the pro-metastatic effects of GDF-15 overexpression are likely more relevant than its tumor-suppressive properties. This is strongly supported by epidemiological data, which shows that GDF-15 is a risk factor for metastasis and death from prostate cancer (83, 85, 100, 184). Overall, the role of GDF-15 in the tumor microenvironment as currently understood, leads to reasonably consistent explanations for the experimental and epidemiological findings on GDF-15 in cancer.

### GDF-15 as a Therapeutic Target for the Treatment of Solid Tumors

The interest in GDF-15 as a target for weight and appetite regulation is reflected by the four independent pharma-driven studies that revealed GFRAL to be the GDF-15 receptor responsible for its metabolic effects (28–30, 41). In consideration of the enormous potential of GFRAL modulators to counteract overeating and obesity, the GDF-15/GFRAL/RET pathway is the most active area for drug development in this endeavor. It would thus be surprising if GDF-15/GFRAL/RET inhibitors were not simultaneously developed for the treatment of cancer. However, the finding that loss of muscle mass depends on GDF-11, whereas GDF-15 is mainly responsible for anorexia, would seem to indicate that such drugs would be of limited usefulness in cancer patients with wasting syndrome (43). Still, anti-GDF-15 antibodies or GFRAL inhibitors may show at least substantial palliative benefit. Treatments that improve the general fitness of cancer patients can also support other treatments (32). Consequently, these findings alone are deemed sufficient to develop GDF-15 as therapeutic target. Another advantage is the ease of measuring GDF-15 serum levels for efficient patient stratification during clinical development (185).

An interesting aspect was added by a recent study demonstrating that β-adrenergic signaling initiated by the GDF-15/GFRAL/RET pathway stimulates the release of tissue-protective hepatic triglycerides (68). It remains to be tested whether this β-adrenergic signaling also protects cancer tissues against the challenges associated with inflammation. If this were indeed confirmed, anti-GDF-15 antibodies or GFRAL inhibitors would have the potential to synergistically boost the effects of chemo-, radiation- or immunotherapy.

Reported tumor suppressor functions of GDF-15 mostly occur during the early phases of tumor development and appear to be restricted to the primary tumor (176). These effects during the early phases of tumor development are of lesser concern for patients who require new treatment modalities due to advanced, metastatic or at least micro-metastatic cancer. Still, it should be explored whether such tumor-suppressive effects can be explained by GDF-15 inhibiting tumor-promoting inflammation, which would be in line with the known functions of GDF-15 and help to provide a conceptual understanding for risk assessment.

The neutralization of the anti-inflammatory functions of GDF-15 could potentially enhance tumor-promoting inflammation. However, converting a non-inflamed into an inflamed tumor would be highly desirable in the context of cancer immunotherapy. Indeed, by inhibiting dendritic cell maturation and by preventing immune cell recruitment, GDF-15 interferes with priming of T cells by dendritic cells and with infiltration of activated T cells into the tumor microenvironment (63, 66, 73, 140). GDF-15 thus impairs two essential steps in the so-called Cancer Immunity Cycle (186). Of note, GDF-15's potential role in the exclusion of T cells from the tumor microenvironment would be consistent with its preferential expression in solid tumors as opposed to hematological malignancies (Figure 2).

The consistently negative correlation between GDF-15 expression and patient survival is consistent with the detrimental impact of immune exclusion on survival (135). Importantly, tumors that are devoid of infiltrating T cells cannot respond to therapies like PD-1 based immune checkpoint blockade (187–189). The conversion of such immune-excluded or immune-neglected “cold” tumors into well-infiltrated, inflamed, “hot” tumors that are accessible for immunotherapies is therefore of significant interest (190). Moreover, as T cells in the tumor microenvironment often acquire an epigenetically imprinted irreversibly exhausted state (191), recruitment of fresh T cells may be generally required to achieve immune-mediated tumor control (189, 191). Since neutralizing GDF-15 could “heat up” the tumor microenvironment, it may already achieve a benefit in monotherapy (135, 181).

Further options arise from GDF-15 inhibition in combination with immune checkpoint blockade, vaccination (192), adoptive transfer of gene-modified T cells (193), and other immunotherapies. In this regard, studies are underway to correlate serum GDF-15 concentrations in cohorts of patients with response or lack of response to PD-(L)1 checkpoint inhibitors. While it is already clear that cancer types with the reportedly highest levels of GDF-15 are less prone to benefit from such single-agent immunotherapies, a possible role for GDF-15 in the evolving landscape of biomarkers for checkpoint inhibitor immunotherapy (194) remains to be established. A recent meta-analysis on biomarker modalities for predicting response to PD-1/PD-L1 checkpoint blockade confirmed that immune cell infiltration is a stronger predictor of response than PD-L1 expression, gene expression profiles or tumor mutational burden (188). The ability of GDF-15 to exclude immune cells from infiltrating a tissue thus warrants further investigation in the context of immune checkpoint inhibition.

Stress-induced GDF-15 production is sufficient to exclude immune cells from the liver (63). This observation could explain why liver metastases are the worst predictors for the success of cancer immunotherapy, as liver metastases hardly ever respond to immunotherapy (195). Neutralization of GDF-15 activity could thus also be a viable approach to extend the benefits of immunotherapy to patients with liver metastases. Overall, there are numerous options for exploring the potential of GDF-15 inhibitors in cancer immunotherapy. As there is no evidence for GFRAL expression in immune cells, the effects of GDF-15 on dendritic cell maturation and immune cell infiltration may be mediated by another still unknown receptor. Thus, it is unclear whether GFRAL inhibitors, which are being developed and tested to treat cancer anorexia (clinical trial identifier NCT04068896), could be a valid alternative to anti-GDF-15 antibodies for immunotherapy.

## Conclusion

GDF-15 is among the most widely overexpressed proteins in human cancer (20). High GDF-15 serum levels are invariably associated with poor prognosis. Functionally, GDF-15 is a main culprit for anorexia in cancer patients (32). Its involvement in muscle wasting has, however, recently been questioned (43). By stimulating hepatic triglyceride release, GDF-15 helps tissues to survive inflammatory stress (68).

Reports on GDF-15-dependent effects in cancer cells are numerous, but the range of reported effects is complex, diverse, and inconsistent, with no consensus in sight. The literature is further marred by artifacts, as a leading commercial source of recombinant GDF-15 was shown to be unfortunately contaminated with bioactive concentrations of TGF-β1 (27).

A more consistent pattern emerges from the literature on the immunomodulatory functions of GDF-15. In line with its presumed physiological role in protecting a semi-allogeneic fetus from the maternal immune system (45), GDF-15 suppresses essential arms of the immune response. Best documented are effects on antigen presentation by dendritic cells (99, 175) and on immune cell trafficking (63, 66, 73, 140). Available data suggest that neutralizing GDF-15 has the potential to not only ameliorate anorexia in cancer patients, but also to improve immunotherapies (108, 140). Given the limited sequence homology between humans and mice, it is not clear whether mouse experiments can fully resolve the still open questions. Based on the clinical correlations and the mechanistic understanding, clinical trials testing anti-GDF-15 treatments appear promising, specifically in patients with elevated GDF-15 serum levels. Considering the mild phenotype of GDF-15 knock-out mice, good tolerability can be expected. Recent disappointing results obtained with combinations of drugs which all aim to rescue the cytolytic effector capacity of T cells (196) further suggest that the targeting of different mechanisms may be required to achieve better synergy. In this regard the “immune cell repellent” GDF-15 which acts at a critical stage of the anti-tumor immune response not addressed by other checkpoints (186) represents a most promising target. Inhibiting an immune evasion mechanism that is potent enough to protect a semi-allogeneic fetus from rejection by the maternal immune system certainly holds potential to improve immune responses against neoantigen-bearing tumor cells (191, 197).

## Author Contributions

JW wrote the article as lead author. IM and WF brought in their specific expertise and contributed to writing as co-authors.

## Conflict of Interest

WF and IM are scientific advisors to Catalym, a biotech company which develops antibodies to GDF-15; JW is a co-founder of Catalym.
